# New Triterpene Diglycosides from the Rhizome of *Cimifuga foetida*

**DOI:** 10.3390/molecules13081712

**Published:** 2008-08-13

**Authors:** Lirong Sun, Jian Yan, Yin Nian, Lin Zhou, Hongjie Zhang, Minghua Qiu

**Affiliations:** 1 State Key Laboratory of Phytochemistry and Plant Resources in West China, Kunming Institute of Botany, the Chinese Academy of Sciences, Kunming, 650204, P. R. China; 2 Graduate School of the Chinese Academy of Sciences, Beijing, 100039, P. R. China; 3 Program for Collaborative Research in the Pharmaceutical Sciences, (m/c877), Department of Medicinal Chemistry and Pharmacognosy, College of Pharmacy, the University of Illinois at Chicago, 833 S. Wood St., Chicago, IL 60612, USA

**Keywords:** *Cimicifuga foetida*, Cycloartane Triterpenoid, Diglycosides, Cimifosides

## Abstract

Five new 9,19-cycloartane triterpene diglycosides, which have been named cimiaceroside C (**1**), and cimifosides A-D (**2**-**5**) together with the known compounds cimiracemoside D (**6**), cimidahurine (**7**) and *α*-d-glucopyranosyl-l-*β*-d-fructofuranoside (**8**) were isolated from the rhizome of *Cimicifuga* *foetida*. The new triterpene diglycosides **1**-**5** were identified as cimiacerol-3-*O*-*β*-d-xylopyranosyl-(1''→3')-*β*-d*-*xylopyranoside, 12*β*-hydroxycimigenol-3-*O*-*β*-d-xylopyranosyl-(1''→3')-*β*-d*-*xylopyranoside, 25-*O*-acetylcimig- enol-3-*O*-*β*-d-xylopyranosyl-(1''→3')-*β*-d*-*xylopyranoside, 24-acetylhydroshengmanol-3-*O*-*β*-d-xylopyranosyl-(1''→3')-*β*-d*-*xylopyranoside and 26-deoxyacetylacteol-3-*O*-*β*-d-xylo- pyranosyl-(1''→3')-*β*-d*-*xylopyranoside, respectively, based on analysis of their spectral data and chemical reactions.

## Introduction

The rhizome of *Cimicifuga* *foetida* (family Ranunculaceae) is a popular Chinese Traditional Medicine. Under the trivial name of “Shengma”, it has been used as an antipyretic and analgesic agent since ancient times [1]. Recently, all *Cimicifuga* species were returned to the genus *Actaea* L based on evidence from DNA sequence data [2,3,4]. In the United States and the European Union, *Actaea* *racemosa* (L.) Nutt [2,3,4], commonly known as black cohosh, has been reputed to reduce the frequency and intensity of hot flashes and other menopause symptoms [5,6]. Chemical constituents of *Cimicifuga* species have been extensively studied by several groups [7,8,9]. Previous phytochemical studies have revealed that *Cimicifuga* species mainly contain constituents such as chromones, cinnamic acid derivatives, and 9,19-cyclolanostane triterpenes. To date, more than 200 triterpenes have been isolated from the genus [10,11] and triterpenoid glycosides are considered to be the main active components, which have been used as marker compounds to standardize *Cimicifuga* extracts [12]. 

**Figure 1:**
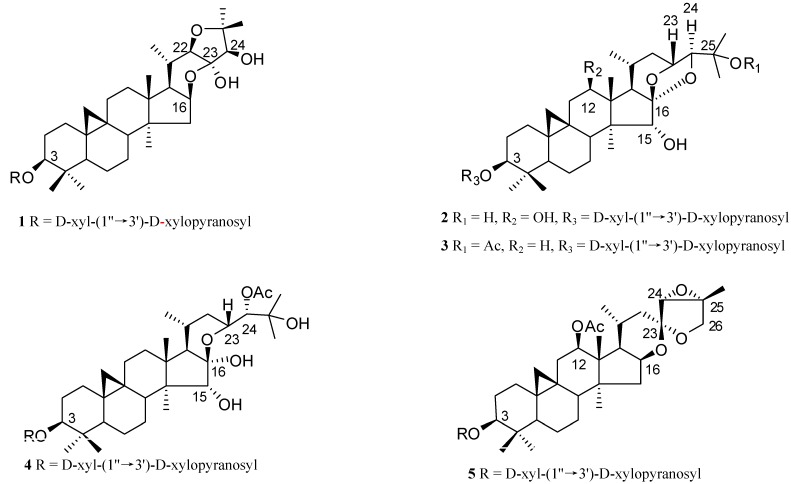
Structures of triterpenoid diglycoside from the *C. foetida.*

In the present study, the constituents of the rhizome of *C. foetida* were investigated. The current paper describes the isolation and identification of the five new cycloartane diglycosides **1-5** (Figure 1), together with the known compounds cimiracemoside D (**6**) [12], cimidahurine (**7**) [13] and *α*-d-glucosyl-*β*-d-fructofuranoside (**8**) [14].

## Results and Discussion

Cimiaceroside C (**1**) was isolated as an amorphous powder. Its molecular formula was determined from its ^13^C-NMR and negative FABMS data (*m/z*: 751 for [M-H]^-^) as C_40_H_64_O_13_, and this was confirmed by negative HRFABMS: *m/z* 751.7352 ([M-H]^-^) (calcd. 751.7362 for C_40_H_63_O_13_). The IR spectrum showed strong hydroxyl bonds at 3376 cm^-1^. The ^13^C-NMR spectrum displayed 40 carbons, which included two sugar moieties at δ_C_ 106.3 (C-1'), 74.6 (C-2'), 87.4 (C-3'), 69.4 (C-4'), 67.5 (C-5'), and at δ_C_ 107.2 (C-1''), 75.4 (C-2''), 78.3 (C-3''), 71.0 (C-4''), 66.6 (C-5'') (Table 1). The ^1^H-NMR spectrum showed the presence of a cyclopropane methylene group at δ_H_ 0.15 and 0.44 (each 1H, d, *J* = 3.6 Hz), seven methyls at δ_H_1.19 (3H, s, Me-18), 1.76 (3H, s, Me-26), 1.67 (3H, s, Me-27), 0.83 (3H, s, Me-28), 1.31 (3H, s, Me-29), 1.02 (3H, s, Me-30) and 1.21 (3H, d, *J* = 6.3 Hz, Me-21), and two sugar anomeric protons at δ_H_5.30 (1H, d, *J* = 7.7 Hz, H-1') and 4.81 (1H, d, *J* = 7.6 Hz, H-1'') (Table 2). The above spectral evidence suggested **1** was a 9,19-cycloartane triterpenoid diglycoside. By acid hydrolysis, only xylose was identified in the aqueous fraction by TLC comparison with an authentic sample, which indicated the both sugar units in **1** were xylose. The NMR spectroscopic data of **1** showed close resemblance with those of cimiaceroside B [15,16], except for presence of an additional sugar unit, which suggested **1** had the same aglycone as that of cimiaceroside B. In the HMBC spectrum, the presence of the long-range correlations of δ_H_ 5.30 (H-1') to 88.6 (C-3), and δ_H_ 4.81 (H-1'') to 87.4 (C-3') determined the inner xylose to be connected at C-3, and the terminal xylose to be at C-3' of the inner xylose. The substituent of the xylose sugar unit at C-3' led to significant downfield shift for the ^13^C-NMR signal at C-3' and upfield shifts for signals at C-2' and C-4'.

**Figure 2:**
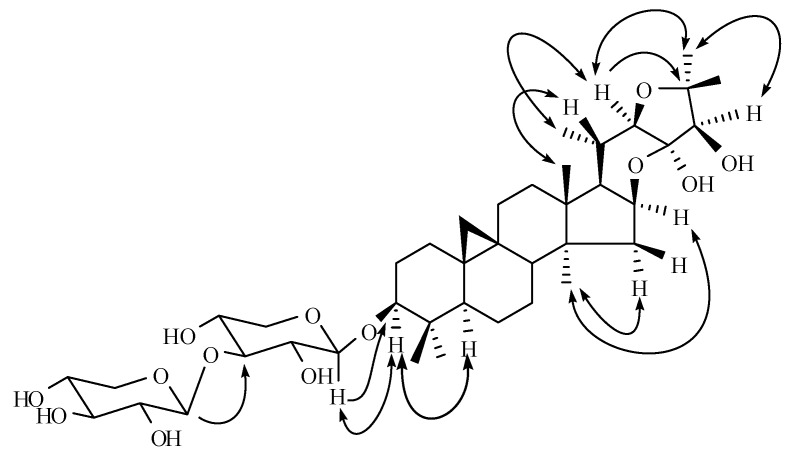
The key HMBC (→) and ROESY (←→) correlations of compound **1**.

The relative stereochemistry of **1** was determined on the basis of the ROESY experiments (Figure 2). A significant ROESY correlation between H-3 and H-5 suggested a *β*-orientation of the substituent at C-3. Moreover, the proton coupling constants of H-1' (J = 7.7 Hz) and H-2'' (*J* = 7.6 Hz) suggested **1** had a *β*-d-xylopyranosyl-(1''→3')-*β*-d*-*xylopyranoside moiety. Thus, cimiaceroside C (**1**) was determined to be cimiacerol-3-*O*-*β*-d-xylopyranosyl-(1''→3')-*β*-d*-*xylopyranoside

**Table 1:** The ^13^C-NMR data of compounds **1**-**5** in C_5_D_5_N (δ in ppm).

Position	1	2	3^a^	4^b^	5^c^
1	32.1 t	32.4 t	32.5 t	32.4 t	31.9 t
2	30.1 t	30.7 t	30.9 t	30.2 t	30.9 t
3	88.6 d	88.7 d	88.8 d	88.5 d	88.3 d
4	41.4 s	41.3 s	41.4 s	41.4 s	41.2 s
5	47.5 d	47.3 d	47.6 d	47.6 d	46.9 d
6	21.0 t	20.9 t	21.1 t	20.5 t	20.2 t
7	26.3 t	26.1 t	26.4 t	26.6 t	25.5 t
8	47.4 d	47.3 d	48.7 d	49.2 d	45.6 d
9	19.7 s	20.8 s	20.0 s	20.1 s	20.1 s
10	26.4 s	26.6 s	26.7 s	26.7 s	26.7 s
11	26.6 t	40.9 t	26.5 t	26.6 t	36.6 t
12	33.5 d	71.8 d	34.1 t	34.1 t	77.1 d
13	46.9 s	47.9 s	41.8 s	42.0 s	48.8 s
14	45.3 s	48.4 s	47.2 s	46.7 s	47.8 s
15	43.3 t	80.0 d	80.2 d	82.8 d	44.1 t
16	72.4 d	112.4 s	112.4 s	103.2 s	74.5 d
17	52.4 d	59.7 d	56.4 d	60.5 d	56.2 d
18	20.7 q	12.1 q	20.0 q	20.1 q	13.5 q
19	30.0 t	30.0 t	30.1 t	30.9 t	29.5 t
20	34.8 d	24.1 d	24.0 d	27.7 d	23.3 d
21	17.5 q	21.1 q	19.5 q	21.4 q	21.3 q
22	86.9 d	38.8 t	38.0 t	34.1 t	37.3 t
23	106.0 s	71.1 d	71.0 d	74.7 d	105.9 s
24	83.3 d	90.1 d	86.8 d	82.7 d	62.5 d
25	83.6 s	71.0 s	83.2 s	71.2 s	62.2 s
26	27.8 q	25.6 q	24.0 q	27.7 q	68.2 t
27	24.9 q	27.1 q	21.5 q	25.3 q	14.3 q
28	19.7 q	11.9 q	11.8 q	12.0 q	19.6 q
29	25.8 q	25.7 q	25.6 q	25.8 q	25.7 q
30	15.5 q	15.4 q	15.5 q	15.5 q	15.3 q
1′	106.3 d	106.3 d	106.3d	106.3 d	106.3 d
2′	74.6 d	74.5 d	74.5 d	74.5 d	74.5 d
3′	87.4 d	87.4 d	87.4 d	87.3 d	87.4 d
4′	69.4 d	69.4 d	69.4 d	69.4 d	69.3 d
5′	67.5 t	67.5 t	67.4 t	67.4 t	67.4 t
1″	107.2 d	107.2 d	107.1 d	107.6 d	107.1 d
2″	75.4 d	75.4 d	75.4 d	75.6 d	75.4 d
3″	78.3 d	78.3 d	78.3 d	78.7 d	78.3 d
4″	71.0 d	71.8 d	71.7 d	71.7 d	71.0 d
5″	66.6 t	66.7 t	66.6 t	67.2 t	66.8 t

The ^13^C-NMR spectral data for the ester moiety: a: δ_C_ 170.2 s (25-COCH_3_), 21.5 s (25-COCH_3_); b: δ_C_ 171.4 s (24-COCH_3_), 21.1 s (24-COCH_3_); c: δ_C_ 170.7 s (12-COCH_3_), 21.6 s (12-COCH_3_)

**Table 2:** The ^1^H-NMR data of compounds **1-5** in C_5_D_5_N (δ in ppm).

Position	1	2	3^a^	4^b^	5^c^
1	1.25 m; 1.52 m	1.15 m; 1.57 m	1.36 m; 1.57 m	1.23 m; 1.51 m	1.13 m; 1.52 m
2	2.25 m; 1.80 m	2.30 m; 1.79 m	1.93 m; 2.34 m	2.29 m; 1.84 m	2.27 m; 1.81 m
3	3.47 dd, 3.8, 11.5	3.45 dd, 3.6, 11.5	3.54 dd, 4.5, 11.5	3.47 dd, 5.0, 9.9	3.40 dd, 4.0, 11.6
5	1.32 m	1.30 m	1.33 m	1.32 m	1.21 m
6	1.15 m; 1.31 m	0.76 dd, 4.5,12.51.60 m	0.66 m; 1.30 m	0.76 dd 4.6,12.5	0.60 m; 1.40 m
7	1.01 m; 1.25 m	1.15 m; 2.10 m	1.12 m; 2.01 m	1.18 m; 2.02 m	0.94 m; 1.22 m
8	1.52 m	1.82 m	1.67	1.90 m	1.56 dd 4.7, 11.5
11	1.25 m	1.54 m	1.06 m; 2.01 m	1.17 m; 2.11 m	1.19 dd 4.4, 13.3;2.68 dd 8.8, 16.0
12	1.51 m	4.20 m	1.54 m; 1.64 m	1.59 m; 1.67 m	5.08 dd 3.2, 8.5
15	1.68 m; 1.90 m	4.47 s	4.14 s	4.26 m	1.78 m; 1.87 dd, 7.9, 12.9
16	4.96 dd 8.0, 16.4	1.78 m			4.21 dd, 7.1, 14.2
17	1.57 m	1.81 m	1.77 m	1.91 m	1.76 m
18	1.19 s	1.41 s	1.22 s	1.28 s	1.39 s
19	0.15 d 3.6; 0.44 d 3.6	0.34 d 3.6; 0.59 d 3.6	0.27 d 4.0; 0.53 d 4.0	0.25 d 2.9; 0.50 d 2.8	0.15 d 3.8; 0.50 d 3.8
20	2.29 m	1.84 m	1.75 m	2.10 s	2.23 m
21	1.21 d 6.3	1.38 d 5.8	0.99 d 5.5	0.82 d 4.9	1.00 d 6.4
22	3.89 d 10.5	1.12 m; 2.38 m	2.04 m; 2.17 m	1.27 m; 2.34 dd 4.6, 11.2	1.45 m; 1.60 m
23		4.77 d 7.9	4.64 d 7.8	4.58 d 7.6	
24	4.18 s	3.82 s	4.10 s	4.14 s	3.64 s
26	1.76 s	1.49 s	1.44 s	1.63 s	3.58 d 10.8; 4.04 d 4.5
27	1.67 s	1.50 s	1.47 s	1.65 s	1.45 s
28	0.83 s	1.21 s	1.30 s	1.18 s	0.82 s
29	1.31 s	1.29 s	1.23 s	1.10 s	1.27 s
30	1.02 s	1.02 s	1.21 s	1.02 s	0.96 s
1′	5.30 d 7.7	5.29 d 7.7	5.32 d 7.6	5.32 d 7.2	5.27 d 7.6
2′	4.10 m	4.12 d 7.7	4.16 m	4.12 m	4.17 m
3′	4.08 m	4.09 m	4.11 m	4.17 m	4.10 m
4′	4.09 m	4.13 m	4.13 m	4.16 m	4.14 m
5′	3.65 m; 4.30 m	3.65 m; 4.31 m	3.65 m; 4.31 m	3.66 m; 4.31 m	3.66 m; 4.32 m
1″	4.81 d 7.6	4.81 d 7.6	4.83 d 7.6	4.80 d 7.6	4.77 d 7.5
2″	4.04 dd 8.2, 16.1	4.04 t 12.7	4.04 d 7.1	4.04 d 7.1	4.06 m
3″	4.11 m	4.17 m	4.13 m	4.16 m	4.16 m
4″	4.12 m	4.20 m	4.13 m	4.13 m	4.13 m
5″	3.68 m; 4.35 m	3.68 m; 4.36 m	3.71 m; 4.36 m	3.68 m; 4.34 m	3.68 m; 4.31 m

The ^1^H-NMR spectral data for the ester moiety: a: δ_H_ 2.01 s (25-COCH_3_); b: δ_H_ 2.11 s (24-COCH_3_); c: δ_H_ 2.11 s (12-COCH_3_).

Cimifoside A (**2**) was isolated as a white powder. The negative HR-FAB-MS of **2** showed a quasi-molecular ion at *m/z* 767.4356, corresponding to the molecular formula of C_40_H_64_O_14_. Its^ 1^H-NMR spectrum (Table 2) exhibited characteristic cyclopropane methylene signals at δ_H_ 0.34 and 0.59 (each 1H, d, *J* = 3.6 Hz), seven methyls at δ_H_1.41 (3H, s, Me-18), 1.49 (3H, s, Me-26), 1.50 (3H, s, Me-27), 1.21 (3H, s, Me-28), 1.29 (3H, s, Me-29), 1.02 (3H, s, Me-30) and 1.38 (3H, d, *J* = 6.3 Hz, Me-21), and two sugar anomeric protons at δ_H_5.29 (1H, d, *J* = 7.7 Hz, H-1') and 4.81 (1H, d, *J* = 7.6 Hz, H-1''). The ^13^C-NMR spectrum exhibited 40 resonances, of which 30 were attributed to a triterpene skeleton, ten to two sugar units. A comparison of the ^1^H- and ^13^C-NMR spectra of **2** with those of cimiside A [17] revealed that **2** has an additional sugar unit. Only xylose was detected in the aqueous fraction of the acid hydrolysis products of **2**. In the HMBC spectrum, long-range correlations between δ_H_ 5.29 (H-1') and 88.7 (C-3), and between δ_H_ 4.81 (H-1'') and 87.4 (C-3') were observed for **2**, which assigned the inner xylose at C-3, and the terminal xylose at C-3' of the inner xylose. The configurations of C(23) and C(24) were ascribed as *R* and *S* respectively, by comparing the spectral data of C-23 and coupling constants of H-24 signals of **2** with those of known 9,19-cyclolanostane triterpene glycosides [18]. Therefore, cimifoside A (**2**) was determined to be 12*β*-hydroxycimigenol-3-*O*-*β*-d-xylopyranosyl-(1''→3')-*β*-d*-*xylopyranoside.

The molecular formula C_42_H_66_O_14_ for cimifoside B (**3**) was established by negative FABMS *m/z*: 793 [M-H]^-^ and HRFABMS 793.4512 (calcd. 793.4573 for C_42_H_65_O_14_). The IR spectrum of **3** showed an absorption at 1739 cm^-1^ due to a carbonyl group. The^ 1^H-NMR spectrum exhibited the cyclopropane methylene signals at δ_H_ 0.27 and 0.53 (each 1H, d, *J* = 4.0 Hz), two anomeric proton signals at δ_H_5.32 (1H, d, *J* = 7.6 Hz, H-1') and 4.83 (1H, d, *J* = 7.6 Hz, H-1'') and a methyl singlet at δ_H_ 2.01 (3H). The ^13^C-NMR spectrum of **3** showed 30 carbon signals for the triterpene skeleton, 10 for two sugar units and two for a carbonyl group. All this evidence suggested that **3** was a 9,19-cycloartane triterpenoid diglycoside. The structure of **3** resembled that of the known compound cimiside B [17]. It differs from cimiside B only by the presence of an acetyl group, which was assigned to C-25 due to dramatic chemical shift changes of C-25 (+Δ12.2 ppm), C-24 (–Δ3.4 ppm), C-26 (–Δ1.4 ppm) and C-27 (–Δ3.9 ppm) (Table 1). The relative configuration of **3 **was determined to be the same as that of **2 **by comparison with literature data [19]. Thus, the chemical structure of **3** was identified to be 12*β*-hydroxycimigenol-3-*O*-*β*-d-xylopyranosyl-(1''→3')-*β*-d*-*xylopyranoside. 

Cimifoside C (**4**) has a molecular formula of C_42_H_68_O_15_ as deduced from the negative HRFABMS (*m/z* 811.4678 [M-H]^-^, calcd. 811.4653 for C_42_H_67_O_15_), together with the ^1^H- and ^13^C-NMR spectra. The IR spectrum of **4** showed absorptions at 3425, 1712 and1266 cm^-1^ due to hydroxyl and carbonyl groups. The^ 1^H-NMR spectrum of **4** exhibited the cyclopropane methylene signals at δ_H_ 0.25 and 0.50 (each 1H, d, *J* = 2.8 Hz), two anomeric proton signals at δ_H_5.32 (1H, d, *J* = 7.2 Hz, H-1'), 4.80 (1H, d, *J* = 7.6 Hz, H-1''), and a methyl signal at δ_H_ 2.11 (3H, s). The ^13^C-NMR spectrum of **4** showed two anomeric carbons at δ_C_ 106.3 (C-1'), 107.2 (C-1'') and a carbonyl group at δ_C_ 171.4 s (24-COCH_3_), and 21.1 s (24-COCH_3_). By comparison of the ^1^H- and ^13^C-NMR spectra with those of 24-acetylhydroshengmanol-3-*O*-*β*-d-xyloside [18], **4** was found to have an additional xylose unit. An HMBC correlation between δ_H_ 4.85 (H-1'') and 87.3 (C-3') determined the terminal xylose to be connected to C-3' of the inner xylose. On the basis of the above evidence, the chemical structure of **4** was assigned as 24-acetylhydroshengmanol-3-*O*-*β*-d-xylopyranosyl-(1''→3')-*β*-d*-*xylopyranoside.

Cimifoside D (**5**) was found to have a molecular formula of C_42_H_52_O_14_ from the ^1^H- and ^13^C-NMR (including DEPT) spectra, which was confirmed by the negative HRFABMS (found 791.4531 for [M-H]^-^, calcd 791.4546 for C_42_H_51_O_14_). The ^1^H- and ^13^C-NMR spectral data of **5** were very similar to those of 23-epi-26-deoxyactin [8], except for an additional xylose. The second xylose was connected to C-3' of the first xylose due to an HMBC correlation between δ_H_ 4.77 (H-1'') and 87.4 (C-3'), and the disaccharide unit was further assigned to C-3 by an HMBC correlation of δ_H_ 5.27 (H-1') and 88.3 (C-3). Hence, **5** was determined to be 23-*epi*-26-deoxyacetylacteol-3-*O*-*β-*d-xylopyranosyl-(1''→3')-*β*-d*-*xylopyranoside.

## Conclusions

Although the rhizome of *C.* *foetida* is a very famous Chinese Traditional Medicine and it has been the subject of extensive phytochemical investigations, its chemical components have not been completely identified yet. Triterpene monoglycosides are considered to be the main components of *Cimicifugu foetida*, but in our present study a series of new cycloartane diglycosides **1-5** have been isolated and identified, which further clarified the triterpene glycoside components from *C.* *foetida*. Moreover this finding will be helpful for identifying extracts of *A.* *racemosa* and *C. foetida*, as some black cohosh products in America and European market are contaminated with related Asian *Cimicifuga* species, such as *C. foetida*, *C. simplex*, and *C. dahurica.*

## Experimental

### General

IR spectra were recorded on a Shimadzu IR-450 instrument, and are reported in cm^-1^. ^1^H (400 and 500 MHz) and ^13^C-NMR (100 and 125 MHz) spectra (all in C_5_D_5_N) were recorded with Bruker AV 400 and DRX500 instruments, using TMS as an internal standard. Silica gel (200-300 mesh, Qingdao Marine Chemical, P.R. China), Lichroprep RP-18 (40-63um, Merck, Darmstadt, Germany) were used for column chromatography (CC). Fractions were monitored by TLC, and spots were visualized by heating TLC sprayed with 10% H_2_SO_4_. Mass spectral data were recorded on a VG Autospec 3000 spectrometer.

### Plant material

The rhizomes of *Cimicifuga foetida* were collected in Lijiang, Yunnan Province, China, in July of 2003 and authenticated by Prof. Zong-Yu Wang (Kunming Institute of Botany, CAS). A voucher specimen (KUN No. 200308025) of the collection has been deposited at State Key Laboratory of Phytochemistry and Plant Resources in West China, Kunming Institute of Botany, the Chinese Academy of Sciences.

### Extraction and Isolation

The dried, milled rhizomes of *C. foetida* (23.4 Kg) were exhaustively extracted with 90% MeOH under reflux. The MeOH extract was evaporated under reduced pressure to yield a syrup (6.2 kg). The syrup was suspended in water-MeOH (9:1), and extracted successively with EtOAc and *n*-BuOH to give an EtOAc extract (1.8 kg) and an *n*-BuOH extract (0.5 kg). The *n*-BuOH extract was subjected to CC (silica gel, CHCl_3_-MeOH 20:1, 10:1 gradient) to yield four fractions (*Fr.*). *Fr.* 1 was rechromatographed on a column (silica gel, CHCl_3_-MeOH 100:1, 80:1, 65:1, 50:1) to obtain **5 **(15 mg), **6** (79 mg) and **7** (36 mg). *Fr.* 2 (1.9 g) was chromatographed repeatedly by CC (silica gel, CHCl_3_- MeOH 10:1) to yield **1** and **2**. *Fr.* 3 was subjected to CC (silica gel, CHCl_3_-MeOH 10:1) and further purified by Sephadex (MeOH) to give **1** (76 mg) and **2** (43 mg). *Fr.* 4 was rechromatographed by CC (silica gel, CHCl_3_-MeOH 5:1, 0:1) to yield crystals of **8** (20 mg).

*Cimiaceroside C* (**1**): white powder; m.p. 292-294 ^o^C; [α]^27^_D_ -31.3° [*c* = 1.60, CHCl_3_-EtOH (1:1)]; negative FABMS *m/z* (%) 751 ([M-H]^-^, 20), 679 (8), 129 (24); negative HRFABMS: *m/z* 751.7352 ([M-H]^-^) (calcd. 751.7362 for C_40_H_63_O_13_); IR (KBr) ν_max_ 3376, 2972, 2940, 2872, 1732, 1639, 1462, 1442, 1382, 1368, 1251, 1209, 1164, 1081, 1052, 1001, 972, 894, 703, 616, 531, 429 cm^-1^; ^13^C- (100 MHz,) and ^1^H-NMR (500 MHz) data, see Table 1 and Table 2.

*Cimifoside* *A* (**2**): white powder; m.p. 287-289 ^o^C; [α]^27^_D_ +16.7° [*c* = 0.60, CHCl_3_-EtOH (1:1)]; negative HRFABMS: *m/z* 767.4366 (calcd. 767.4342 for C_40_H_6__4_O_1__3_); IR (KBr) ν_max_ 3431, 2928, 2870, 1631, 1455, 1383, 1363, 1170, 1077, 1040, 563, 470 cm^-1^; ^13^C- (100 MHz,) and ^1^H-NMR (500 MHz) data, see Table 1 and Table 2.

*Cimifoside B* (**3**): white powder; m.p. 165-166 ^o^C; [α]^27^_D_ -24.2° [*c* = 0.85, CHCl_3_-EtOH (1:1)]; negative HRFABMS: *m/z* 793.4512, (calcd. 793.4573 for C_42_H_65_O_14_,); IR (KBr) ν_max_ 3443, 2963, 2934, 2870, 1739, 1735, 1457, 1239, 1070 cm^-1^; ^13^C- (100 MHz,) and ^1^H-NMR (500 MHz) data, see Table 1 and Table 2.

*Cimifoside* *C* (**4**): white powder; m.p. 224-225 ^o^C; [α]^27^_D_ -60.0° [*c* = 1.50, CHCl_3_-EtOH (1:1)]; negative HRFABMS: *m/z* 811.4678, (calcd. 811.4653 for C_42_H_67_O_15_); IR (KBr) ν_max_ 3425, 2940, 2870, 1712, 1632, 1457, 1379, 1266, 1159, 1107, 1072, 1042, 996, 969, 898, 797, 738, 630, 610, 591, 496, 449 cm^-1^; ^13^C- (100 MHz,) and ^1^H-NMR (500 MHz) data, see Table 1 and Table 2.

*Cimifoside* *D* (**5**): white powder; m.p. 185-186 ^o^C; [α]^27^_D_ 56.2° [*c* = 0.76, CHCl_3_:EtOH (1:1)]; negative HRFABMS: *m/z* 791.4531([M-H]^-^) (calcd. 791.4546 for C_42_H_53_O_14_); IR (KBr) ν_max_ 3442, 2969, 2937, 2872, 1731, 1633, 1458, 1382, 1366, 1252, 1165, 1042, 984, 898, 841, 804, 677, 629, 602 cm^-1^; ^13^C- (100 MHz,) and ^1^H-NMR (500 MHz) data, see Table 1 and Table 2.

### Acid hydrolysis of compounds **1-5**

Compounds **1**-**5** (6 mg of each) were refluxed with 5% HCl in MeOH (7 mL) for 8 h. Each mixture was diluted with H_2_O and neutralized with NaHCO_3_. The neutral hydrolysate revealed the presence of only xylose by TLC (n-BuOH-AcOH-H_2_O, 4:1:1, *Rf* = 0.4) upon comparison with the authentic sample.
